# Outcome of childhood acute lymphoblastic leukemia treatment in a single center in Brazil: A survival analysis study

**DOI:** 10.1002/cnr2.1452

**Published:** 2021-06-11

**Authors:** Thais A. Bonilha, Danielle D. A. Obadia, Andressa C. Valveson, Marcelo G. P. Land

**Affiliations:** ^1^ Department of Pediatrics Instituto Estadual de Hematologia Arthur de Siqueira Cavalcanti – Hemorio Rio de Janeiro Brazil; ^2^ Clinical Medicine Post‐Graduation Program, College of Medicine Universidade Federal do Rio de Janeiro Rio de Janeiro Brazil; ^3^ Department of Pediatrics, Instituto de Puericultura e Pediatria Martagão Gesteira Universidade Federal do Rio de Janeiro Rio de Janeiro Brazil

**Keywords:** acute lymphoblastic leukemia, asparaginase, AYA, pediatrics, prognostic factors

## Abstract

**Background:**

Acute lymphoblastic leukemia (ALL) is the most common neoplasm in childhood. The probability of current overall survival (OS) is around 90% in developed countries. There are few studies that demonstrate the results in Brazil.

**Aim:**

This work aims to analyze the results of children with ALL treated at a single institution in Rio de Janeiro.

**Methods and results:**

Retrospective analysis survival study of a cohort of childhood ALL patients treated in Hemorio. Kaplan–Meier and log‐rank methods were used for the analysis of OS and events‐free survival (EFS) and the Cox proportional hazards regression model for multivariate analysis.

The probability of OS and EFS at 6 years was 52% and 45%. The probability of OS and EFS in 6 years for patients aged 10‐17 years was 31% and 28% and for the younger was 65% and 55%, respectively (*p* < .001). A probability of OS and EFS in 6 years for patients with more than 100 000 leukocytes/mm^3^ at diagnosis was 19% and 16% and those with less than 100 000 were 62% (*p* = .007) and 55% (*p* = .008). Those who received less than 10 doses of native *Escherichia coli* asparaginase had a probability of OS and EFS in 6 years of 27% and 21% and those who received at least 10 doses were 74% and 65% (*p* < .001).

**Conclusions:**

The presence of a high number of adolescents and high‐risk patients, as well as many patients who discontinued the use of asparaginase or any substitute led to a lower probability of OS and EFS in our cohort.

## 1 INTRODUCTION

Acute lymphoblastic leukemia (ALL) is the most common neoplasm in children, corresponding to 25% of all childhood cancers and it is the main cause of death related to cancer in this population.^1^, ^2^, ^3^ About 3000 new cases are estimated in the United States^2^ and the incidence peak is 2‐5 years.^4^ ALL is more common in male Caucasians, and also more frequent in developed countries and urban areas.^5^

Better understanding and identification of prognostic factors and risk stratification leads to a more appropriate treatment of ALL. Patients aged 1‐9 years had favorable outcomes when compared with those between 10 and 17 years and infants <1 year. These three groups had different clinical and biological features.^6^, ^7^ The initial white blood cell count (WBC) is also an important clinical characteristic. Patients with more than 50 000 WBC/mm^3^ at diagnosis had a worse outcome than those who had lower initial WBC.^5^, ^8^ Considering immunophenotype, T‐ALL was previously considered to have the worst prognosis. However, with more intense chemotherapy protocols, especially with high doses of methotrexate, dexamethasone, and asparaginase, the results of this group became similar to B lineage ALL.^9^, ^10^ Despite that, T‐ALL still have a higher risk of induction failure, central nervous system (CNS) relapse, and early relapse.^7^

Early response to treatment is the most important prognostic feature in childhood ALL. For patients who presented minimal residual disease (MRD) by flow cytometry on D15 ≥ 10%, a probability of 5‐year of event‐free survival (EFS) was 46.1% while for those with MRD < 0.1% it was 89.9% (*p*‐value < .001).^11^ A positive MRD at the end of induction and consolidation is also associated with more risk of relapse.^12^ The number of blasts in peripheral blood after 7 day of prednisone was utilized for risk stratification in many protocols, however, the poor response to prednisone lose its importance in B lineage ALL after the introduction of MRD determination on D15.^11^

Recently, advances in treatment and improvements in life support led to a probability of 5‐year overall survival (OS) of nearly 90%.^2^, ^3^, ^5^, ^13^, ^14^ During the year 2014 the Intercontinental Berlin–Frankfurt–Muenster group (IC‐BFM) published the results of 5060 patients enrolled in the ALL‐IC‐BFM 2002 study where a probability of 5‐year OS and EFS of 82% and 74% was observed. The patients were classified into three risk groups: standard (SR), intermediate (IR), and high (HR). The SR group had a probability of 5‐year OS of 90% while for IR and HR it was 86% and 62%, respectively.^15^ The results of Children's Cancer Group (CCG) and Children's Oncology Group (COG) clinical trials between 2006 and 2009 were similar to IC‐BFM group. They showed a probability of 5‐year OS of more than 90% in a study with 6530 patients.^6^

There are few studies in Brazil that demonstrated if these good results could be obtained in our country (a middle‐income country) and in low‐income countries. The present study has the intention to analyze the outcomes of children and adolescents treated in one of the most important institutions for the treatment of hematological diseases in Rio de Janeiro.

## 2 PATIENTS AND METHODS

This is a retrospective survival analysis study performed from January 2011 to December 2017 and includes children and adolescents with ALL, aged 1‐17 years that were treated in Arthur de Siqueira Cavalcanti State Institute of Hematology in Rio de Janeiro, Brazil with ALL IC‐BFM 2002 based protocol.^16^ Patients with mature B cell ALL were excluded.

ALL was diagnosed when ≥25% of lymphoblasts were present in the bone marrow. Immunophenotyping by flow cytometry, cytogenetic, and molecular analysis was performed according to the ALL IC‐BFM 2002 protocol. All patients were stratified into three risk groups and treated according to this classification. Due to limited resources or the unavailability of some drugs in our country, accommodations had to be performed to the protocol:

1. Replacement of vindesine with vincristine.
2. Not all patients were able to receive 5000 mg/m^2^ of metotrexate, most patients had to receive 2000 mg/m^2^.
3. Citarabine was administrated subcutaneously in phase 2 of protocol I and II.
4. Replacement of prednisolone with dexamethasone in intrathecal therapy.

Data were analyzed using R software. EFS was defined as the time from diagnosis to the date of the first event. The considered events were relapse, death, or second malignant neoplasm. OS was calculated from the date of diagnosis to date of death of any cause or to the date of the last follow‐up. EFS and survival curves were calculated with Kaplan–Meier survival analysis and the curves were compared by log‐rank test. For multivariable analysis was used the Cox proportional hazard regression model. Proportional hazards assumption was tested by Schoenfeld residuals. The Martingale residuals plot was used to determine the optimal cutpoint for the number of native asparaginase doses variable. Variables with 80% significance (*p*‐value < .2) were selected for multivariable analysis. After the assessment of the multicollinearity among every possible candidate variable, a parsimonious model was built through the backward elimination method, using the likelihood ratio test as selection criteria.

## 3 RESULTS

A total of 100 patients were eligible for this study. There were no losses. Their clinical characteristics are summarized in Table 1. The median age was 8.3 and the ratio of male to female was 1.22:1.

**TABLE 1:** Patient's characteristics

	Number (%)	Median
**Age (years) (*N* = 100**)	100	8.3 (1.3–17.8)
≥ 1 e < 10	63 (63)	
≥ 10	37 (37)	
**Gender (*N* = 100)**		
Male	55 (55)	
Female	45 (45)	
**Final risk (*N* = 97)**		
Standard	14 (14)	
Intermediate	28 (29)	
High	55 (57)	
**Initial WBC** ^a^ **(×10** ^**3**^ **/L) (*N* = 98)**		18 100 (600–520 000)
<50	60 (61.3)	
50 a 100	18 (18.3)	
>100	20 (20.4)	
**Immunofenotype**		
B	75 (75)	
T	25 (25)	
**CNS** ^b^ **status (*N* = 100)**		
Positive	12 (12)	
Negative	88 (88)	
**Deaths before CR** ^c^	3 (3)	
**Relapses**	32 (32)	
**Cytogenetics (*N* = 65)**		
Hyperdiploid	4 (6.2)	
*t*(1;19)	2 (3.1)	
46XX or 46XY	10 (15.4)	
No mitosis	46 (70.7)	
Others	3 (4.6)	
**Molecular biology (*N* = 65)**		
t(9;22)/BCR‐ABL	10 (15.4)	
t(1;19)/E2A‐PBX1	4 (6.2)	
t(12;21)/TEL‐AML	6 (9.2)	
Negative	45 (69.2)	

^a^ White blood cell.

^b^ Central nervous system.

^c^ Complete remission.

**FIGURE 1:**
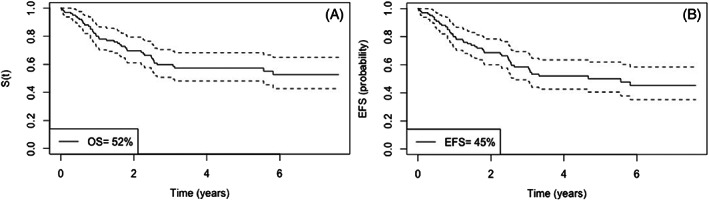
(A) Kaplan–Meier curve of overall survival. (B) Kaplan–Meier curve of events free survival

For the entire cohort, the probability of 6‐year OS was 52% while the probability of EFS was 45% (Figure 1A,B). There were three deaths during the induction and the main cause of death in all phases of treatment was infection (48%). A total of 32%of the patients relapsed.

**FIGURE 2:**
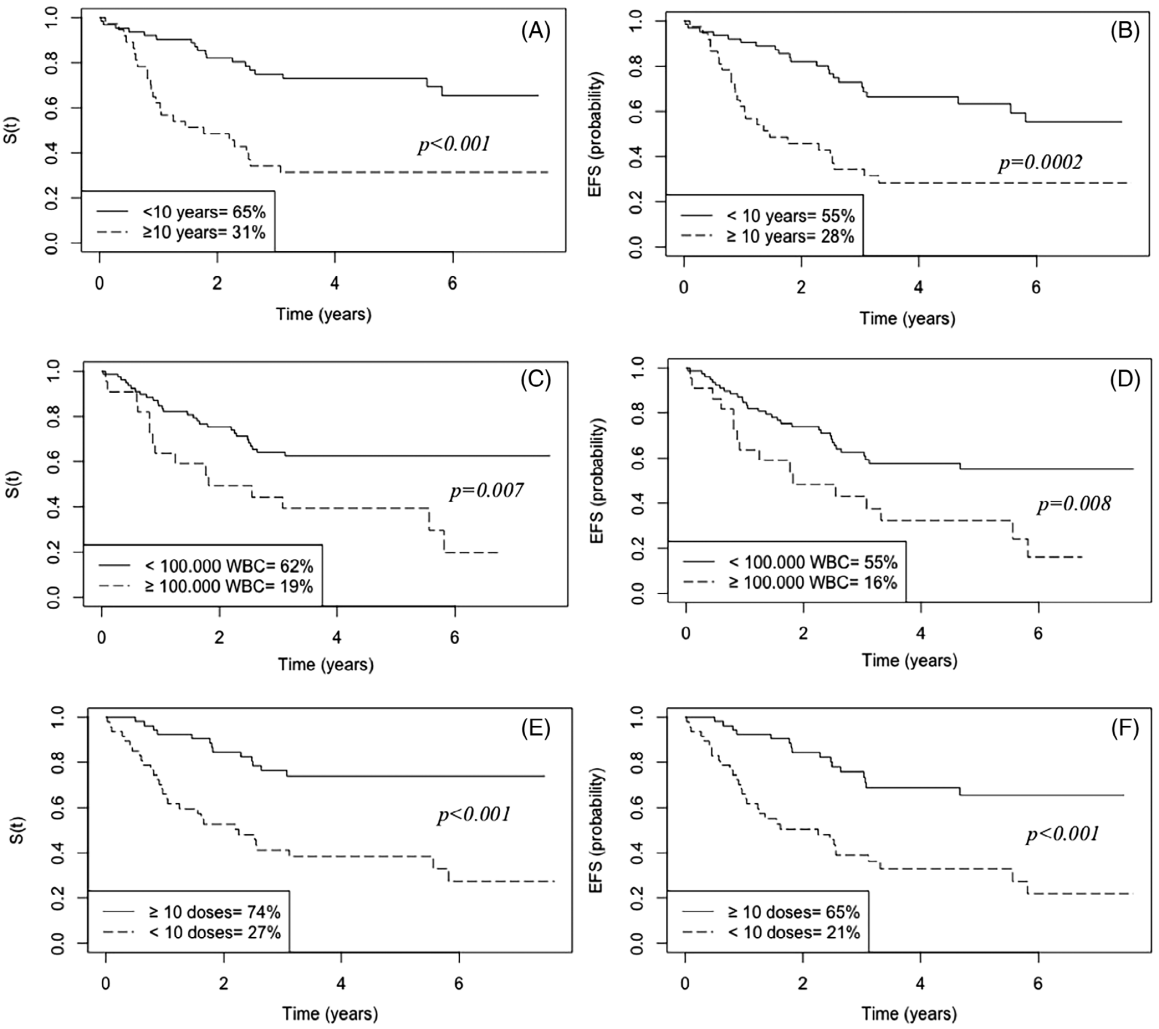
Kaplan–Meier curve of overall survival by age (A), initial white blood cell (C) and number of doses of native *Escherichia coli* asparaginase received (E) and Kaplan–Meier curve of events free survival by age (B), initial white blood cell (D) and number of doses of native *E. coli* asparaginase received (F)

Outcomes in adolescents (10‐17 years) as compared to children (1‐9 years) were significantly worse: 6‐year EFS was 28% versus 55% (*p*‐value = .0002) and 6‐year OS was 31% versus 65% (*p*‐value < .001) (Figure 2A,B). The 6‐year EFS and OS were 16% and 19% for those with more than 100 000 WBC versus 55% and 62% for those with less than 100 000 WBC (*p*‐value = .008 and .0066) (Figure 2C,D). Another important prognostic factor in this study was the number of doses of native *Escherichia coli* asparaginase effectively received. The patients who received less than 10 doses had a worse outcome as compared to those who received 10 or more doses: 6‐year EFS was 21% versus 65% (*p*‐value < .001) and 6‐year OS was 27% versus 74% (*p*‐value < .001) (Figure 2E,F).

The patients were classified into different risk groups according to ALL IC‐BFM 2002 protocol. The 6‐year EFS and OS for the SR group were 69% and 74%, for the IR group were 47% and 57% while for the HR group were 38% and 42%. The better survival probability of the standard group was statistically significant when compared to the HR (*p*‐value = .045).

When analyzed by immunophenotype, the probability of 6‐year EFS was 26% for T‐ALL versus 52% for pB‐ALL (*p*‐value = .02) while the probability of 6‐year OS was 27% versus 61% (*p*‐value = .0067).

A Cox proportional hazard regression model was used to analyze the hazards of death and events. In the univariate analysis, patients aged between 10 and 17 years, initial WBC > 100 000, T cell ALL and less than 10 doses of native *E. coli* asparaginase received, had a major hazard for death or relapse. In this analysis we found that be classified as SR was a protector factor. For multivariate analysis, we use all variables that had a *p*‐value < .2 and this way we could found the most important prognostic features related to worse outcomes in our cohort (age, initial WBC, and the number of doses of native *E. coli* asparaginase received).

No statistical difference was found when analyzed the groups by sex, skin color, or CNS status.

## 4 DISCUSSION

In the present study, we found a total of 3 deaths before the complete remission that was lower than other results in Brazil (5.5% in Pernambuco, 6.8% in GBTLI‐Brazilian Cooperative Group for Childhood ALL Treatment—ALL‐93 study and 22% in Sergipe).^17^, ^18^, ^19^ and similar to the results found in the ALL IC‐BFM 2002 protocol (2.1%).^15^

Our cohort had a median age of 8.3 years that is older than the incidence peaks of ALL between 2 and 5 years..^5^, ^6^ There were a higher number of adolescents 10‐17 years as compared with the AIEOP‐BFM ALL 2000 (37% vs. 23%) and the ALL IC‐BFM 2002 studies (37% vs. 26%).^15^, ^20^ Our data confirm the worse outcome for the ALL patients older than 10 years when compared to the younger's one: the probability of 6‐year EFS and OS were 28% and 31% for the adolescents versus 55% and 65% for the children.

The adolescents are often classified as an HR group and for this reason, usually receive more intense chemotherapy. This more intensive treatment leads to a higher incidence of treatment‐related complications and more probability of severe infections, which was our major cause of deaths (almost 50%). The adolescent also has worse clinical features as more frequency of T‐ALL immunophenotype, higher WBC at diagnosis, poor treatment response, the absence of *ETV6/RUNX1*, and the presence of *BCR/ABL1* fusion genes.^20^ This genetic feature was found in 15% of the patients who were able to realize the molecular analysis, a number much higher than the literature,^21^ what could not be explained only by the number of adolescents. The low number of patients in this cohort limits a better analysis of that found.

This study had a high number of patients classified as HR (57%) and only 14% as SR. This data is much different from the ALL IC‐BFM 2002 study, which had 16.7% of HR and 30% of SR.^15^ Analyzing the results for each risk group, only the OS of SR patients was similar compared to IC‐BFM group. The probability of 6‐year OS was 85% versus 90% in ALL IC‐BFM 2002 study.^15^ For the HR patients, the difference between the two studies was huge. While in the present study the probability of 6‐year EFS and OS were 38% and 42%, in the ALL IC‐BFM 2002 study it was 55% and 62% in HR.^15^ The difference between the number of patients classified as HR could explain the lower probability of EFS and OS find in our cohort. However, such difference is not the only responsible for our worse outcome, since it persists in all risk groups. The fact that we had many patients who could not receive all doses of asparaginase adds to that. Also, we have many patients who did not get 5 g/m^2^ of methotrexate what could contribute to a worse outcome.

When compared this study to others in Brazil, we found more similarities in the results. Laks et al published in 2003 that a probability of 5‐year OS was 56.5% in a cohort from Rio Grande do Sul.^22^ The GBTLI ALL‐93 study showed a probability of 15‐year EFS of 53.8% in HR patients.^19^ Lustosa de Sousa et al in 2015 described the results of a cohort from 3 hospitals in Ceará. On their study all patients have treated with GBTLI‐ALL 93 and 99 e the probability of 5‐years OS 72%,^23^ however, they only had 22.4% of patients more than 9 years. More recently, Pedrosa et al demonstrated that the probability of 5‐year EFS and OS were 92% and 96% in patients treated with RELLA05 protocol.^24^ These data are referring only to patients with very low risk (pB‐ALL, aged 1‐10 years, initial WBC <50 000, MRD <0.01% in D19 from induction and without extramedullary involvement) what explain the better results obtained by their group.

The initial WBC was an important prognostic feature according to the literature. Patients with more than 100 000 WBC at diagnosis had a probability of 6‐year EFS of 16% versus 55% (*p*‐value = .008) in those who had less than 100 000 initial WBC, while the probability of 6‐year OS was 19% versus 62% (*p*‐value = .0067). When compared to the studies of BFM group (IC‐BFM ALL 2002 and AIEOP‐BFM ALL 2000) this data had shown a higher number of patients who presented with hyperleukocytosis (>100 000), 20% versus 11.3% and 9.9%.^15^, ^20^ A high initial WBC is also found more frequently in adolescents.

Another important prognostic feature was the number of doses of asparaginase. Patients who received at least 10 doses had a probability of 6‐year EFS and OS of 65% and 74% versus 21% and 27% in those who received less than 10 doses (*p*‐value < .001). In multivariable analysis, the group who received fewer doses of asparaginase had a hazard ratio of 2.56 times for death compared with the other group. The number of doses of asparaginase had a great impact on OS and EFS because had a lot of patients had to discontinue the use of this medication. A total of 47% of patients received less than 10 doses. Dana‐Farber Cancer Institute utilizes a treatment with a prolonged depletion of the amino acid asparagine showed the importance of asparaginase in the outcome of ALL patients. Silverman demonstrated with this protocol that the patients who received 25 weeks of asparaginase or less had a worse probability of 5‐year OS than the group who received at least 26 weeks of medication (73% vs. 92%, *p*‐value < .01).^25^ COG also publishes a study that confirms the importance of discontinuing asparaginase in the treatment of ALL. The results obtained in this study reveal that the group who discontinues peg‐asparaginase without substitution by erwinase had a hazard ratio 1.5 times for the evaluated events and the group who got erwinase do not have any differences in EFS.^26^

In conclusion, it was not possible to repeat the good outcomes obtained by BFM group. The results of this study confirm the importance of age, initial WBC, and number of doses of asparaginase on the outcome of ALL pediatric patients. The low number of patents and the nature of the study limits some of the results. The most important prognostic features were age 10‐17 years, initial WBC >100 000, and less than 10 doses of asparaginase received. We can see that the results were influenced by the socio‐economic situation of our country and by the failures of the public health system since the main points that we need to improve to obtain better outcomes are improvement the life support; make erwinase available for those who could not receive asparaginase and follow the treatment protocol in its entirety.

## CONFLICT OF INTEREST

The authors declare no potential conflicts of interest.

## AUTHOR CONTRIBUTIONS

*Conceptualization; data curation; formal analysis; investigation; methodology; project administration; visualization; writing‐original draft; writing‐review & editing*, T.A.B.; *Data curation; investigation*, D.D.A.O. and A.C.V.; *Conceptualization; data curation; formal analysis; investigation; methodology; project administration; supervision; validation; visualization; writing‐original draft; writing‐review & editing*, M.L.

## ETHICS STATEMENT

The presented study was approved by the Hemorio Ethics Committee on November 29, 2018 under the registration number 445/18. Since this was a retrospective study that did not generate any intervention, the free and informed consent term was waived.

## Data Availability

The data that support the findings of this study are available from the corresponding author upon reasonable request.
